# Diversity of Mosquito Vectors (Diptera: Culicidae) in Caxiuanã, Pará, Brazil

**DOI:** 10.1155/2012/741273

**Published:** 2012-09-11

**Authors:** Ulisses E. C. Confalonieri, Cristina Costa Neto

**Affiliations:** ^1^Fiocruz, CPqRR, LAESA, Avenue Augusto de Lima, 1715, sala 206, 30190-002 Belo Horizonte, MG, Brazil; ^2^PMAGS, DCB, ENSP Expansão do Campus, Avenue Brasil, 4036 sala 703, 21040-361 Rio de Janeiro, RJ, Brazil

## Abstract

This paper presents a study based on ecological parameters represented by diversity and richness indices applied in a community of mosquitoes (Diptera: Culicidae), at the National Forest of Caxiuanã, Melgaço municipality, state of Pará, in the Brazilian Amazon. A total of 25,433 specimens of culicids were collected in the study, from five field collection periods, over 10 months, between 2005 and 2006. Specimens were collected in four heights of the forest (ground level, 8 m, 16 m, and 30 m-canopy). Diversity indices of Shannon and Berger-Parker were obtained, and indicators of dominance of species were calculated. The species *Culex portesi* was dominant in this site, representing about 84% of specimens. Measures of richness and similarity (Jaccard) were obtained for the five strata of time and four height levels. According to the richness estimator abundance-based covered estimator (ACE) the greatest value occurred in April (2006), considering the levels of height to 16 m and on the ground. The estimates obtained have shown quantitative parameters of mosquito populations in the region of the Forest of Caxiuanã.

## 1. Introduction

 Ecologists and biologists measure the biological diversity of a given region or site for several reasons but usually to study the ecological and evolutionary processes and to analyze changes in diversity, similarity, and dominance of species in a time scale [1].

 Although extensively used in community ecological studies of several groups of insects [2–4] only in the past ten years similarity studies for communities of mosquito species vectors of pathogens in the Neotropics were performed, as discussed elsewhere [5–11].

 However, the most recent field surveys of mosquito populations in the Amazon region did not analyze species richness/diversity using quantitative estimates, such as those of Hutchings [12–14], Santos et al. [15], Jones et al. [16], Fé et al. [17], Mondet et al. [18] and Souto [19].

 In this study we have collected mosquito samples (Diptera: Culicidae) in a pristine rainforest of the Amazon at four different vertical strata of the forest and at different times of the year, during a period of a 10 months. Quantitative estimates of diversity and similarity were obtained using different indicators currently in use.

## 2. Materials and Methods

 Mosquito samples were collected at the National Forest of Caxiuanã, municipality of Melgaço, state of Pará, Brazil. Field trips were performed in the months of July, September, and December 2005 and February and April of 2006. The study site was a meteorological tower (54 meters high) of the project LBA-Large Scale Biosphere-Atmosphere Experiment in the Amazon. It is a pristine forest ecosystem at the Ferreira Pena Research Station of the Museu Paraense Emilio Goeldi, Brazilian Ministry of Science and Technology. A total of 25,433 specimens were collected at four different heights in the tower: ground level (0 m), 8 m, 16 m and 30 m (canopy level). Details and duration of the collection, techniques, preparation, and identification of the entomological material were published elsewhere [20, 21]). In summary, in each of the five field trips three night-time (6:00 pm–6:00 am) and two diurnal (6:00 am–6:00 pm) collecting periods lasting twelve hours each were performed. Mosquito captures were conducted using CDC light traps as well as entomological hand nets at four different heights: 30 m (forest canopy), 16 m, 8 m, and ground level. One collector and one CDC trap were placed at each height. Specimens were identified using the keys provided by Consoli and Lourenço-de-Oliveira [22] and Forattini [23].

 Species diversity indices were obtained as well as nonparametric richness estimators for the differences in diversity in the different heights of collection and different times of the year. Rarefaction curves were also obtained (species accumulation in relation to the collecting efforts), with the aim of observing the asymptotic trends of the number of species in the strata and for an evaluation of the similarity both in the temporal (collection periods) and spatial (heights) levels.

 To verify the sampling sufficiency to assess richness, directly related to the number of rare species in the samples, nonparametric estimates were obtained: Chao 1; Bootstrap and Abundance-based Coverage Estimator (ACE). The indices of diversity were calculated using the software PAST [24], under public domain.

 For the analysis of similarity among the samples from different heights and periods of collection (time of the year) the Jaccard Index was used [1].

## 3. Results

 The total number of culicid specimens collected was 25,433, reaching a total of 15 genera and 55 species. [25] as shown in Table 1.

The diversity indexes describe the parameters of diversity that characterizes the region studied. On the first line, the number of species was found at this site, in each time of the collection. The number of specimens, is the number of mosquitoes found in each time of collection. Below comes the diversity indexes; Berger-Parker is an indicator of dominance of the sample collected at each time, the greater the index, higher is the dominance of a species. Shannon *H*′ is a measure of the diversity itself, shown in a comparative way. Evenness (*E*) is a measure of how much the species are equal in the sense of number of specimens, for each time of collection. It is a similar measure to equitability, and it is given by *E* = *e* ^∧^ *H*′/*S* where *H*′ is the observed diversity index, and *e* is the base of the natural logarithms. The quantity *e* ^∧^ *H*′* * is the minimum number of equally common species which could yield the observed diversity *H*′. The equitability (*ε*) concept [26, 27] is based on the assumption that *ε* = *S*′/*S*, where *S*′ is the theoretical number of species which would yield the observed diversity *H*′ if their relative abundances followed the broken-stick model of MacArthur.

The diversity indices for the temporal samples of the culicids collected are depicted in Table 2.

Species richness (i.e., the number of species) is the simplest and the most intuitive concept for characterizing community diversity. We focus on the estimation of species richness based on a sample from a local community. The topic is important for comparing communities in conservation and management of biodiversity, for assessing the effects of human disturbance on biodiversity, and for making environmental policy decisions [28]. The compilation of complete species census and inventories often requires extraordinary efforts and is an almost unattainable goal in practical applications. There are undiscovered species in almost every taxonomic survey or species inventory. In the next tables of species richness estimates we present nonparametric approaches which avoid making assumptions about species discovery rates. (1) Estimator by Chao (*S*_Chao1_) is based on the concept that rare species carry the most information about the number of missing ones and used only the singletons (*f*1) and doubletons (*f*2) to estimate the number of missing species. The use of this estimator as a point estimator has been recently justified under practical assumptions. (2) Bootstrap method: given the *n* individuals who were already observed in the experiment, draw a random sample of size *n* from these individuals with replacement. Assume that the proportion of the individuals for the *i*th species in the generated sample is ${\hat{h}}_{i}$. Then a bootstrap estimate of species richness is calculated by the formula $\hat{S}=S+\Sigma_{i=1}^{S}{\left(1-{\hat{h}}_{i}\right)}^{n}$. After a sufficient number of bootstrap estimates are computed, their average is taken as a final estimate. (3) Abundance-based coverage estimator (*S*_ACE_): the approach separates the observed frequencies into two groups: abundant and rare. A value of the cut-off point of *k* = 10, where *k* is the number of specimens, is suggested based on empirical evidence. The exact frequencies for the rare species are required because the estimation of the number of missing species is based entirely on these frequencies.

In Table 3 the species richness estimates for the temporal samples (periods of field collections) are presented [29].

The results below show the same diversity indexes as explained above, for the four strata of heights, ground (0 meters), 8 meters, 16 meters, and 30 meters (canopy level). Observe that the community is the same, only rearranged by heights of collection. When we consider the strata of time, it includes all the four heights, and when we consider the strata of heights, this includes all the five time periods of collection.

Table 4 shows the mosquito diversity Indices obtained for the four different heights in the forest, and Table 5 depicts the *t*-test for the Shannon Indices.

In Table 5 below, we make a statistical significance test for the difference in diversity for the four levels of heights. This test was made in PAST [24]. We only test consecutive heights.

The richness estimates according to the different forest strata are shown in Table 6.

Rarefaction curves are a technique to assess species richness from the results of sampling. This curve is a plot of the number of species as a function of the number of samples. On the left, the steep slope indicates that a large fraction of the species diversity remains to be discovered. If the curve becomes flatter to the right, a reasonable number of individual samples have been taken; more intensive sampling is likely to yield only few additional species.

The rarefaction curves for spatial (heights) samples can be observed below.

We have also evaluated the species similarity among the samples using the Jaccard statistic, both for the periods of the year and the forest heights. These are shown in Tables 7 and 8. The similarity Jaccard index measures how much two communities (A and B) have in common (it is calculated by (A∩B/A ∪ B)). In our case, we consider similarities between strata within the same community, of time periods and of heights.

## 4. Discussion

 Rare species, as shown by the tail of the distribution graphs for abundance, have a strong influence on the estimates of diversity, especially those related to species richness. It has also an influence on the Shannon evenness index, as shown by the inclination of the straight line, related to degrees of abundance, in the geometrical model graph. Although the sample for December 05 has the smallest number of species, as compared with the other temporal samples, it has a high number of “singletons”, and the Chao estimate for richness has indicated a higher number of species. In a similar way, although the strata 8 m, 16 m, and 30 m did not show much difference in the number of species, the Chao 1 estimator has indicated a larger number of species for the 16 m height due to its high number of singletons.

 Some of the diversity indices were developed to estimate community parameters but the hypothesis for the occurrence of a nonbiased estimate can be restrictive, and the parameters sometimes may be difficult to use [28]. As an example we have the Shannon index which assumes that all specimens collected come from an infinitely large population and that all species are represented in the sample. Diversity indices are also influenced by sample sizes and sampling methods and, therefore, are limited for estimating community parameters, but they are useful for the identification of differences among sampled groups. According to Taylor and Bates [30] diversity indices are as good as their capacity to discriminate among the groups. On the other hand, diversity indices such as the Berger-Parker and Simpson—which have an inverse relationship with diversity—are strongly influenced by the dominant species.

 In our work we have obtained a Shannon (*H*′) index of 0.34 for September 05 and 1.23 for July 05; after the transformations these Indexes were, respectively, exp(0.34) = 1.41 and exp(1.23) = 3.42. These values indicate a low number of species, probably associated with the high dominance of the species *Culex portesi.*

**Figure 1:**
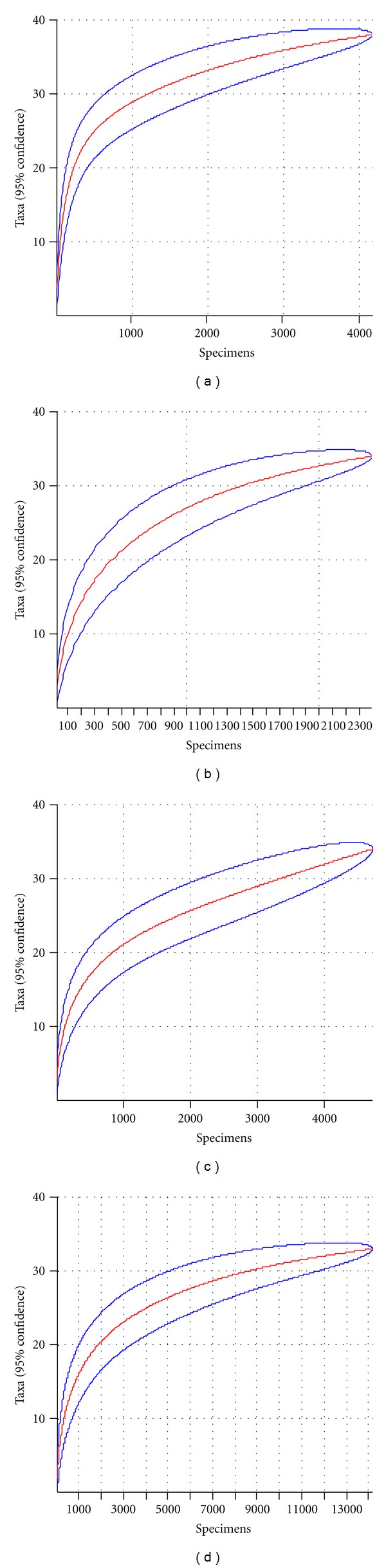
Rarefaction curves (accumulation of species) for different forest strata or spatial levels: (a) ground level, (b) 8 meters, (c) 16 meters, and (d) 30 meters (canopy level).

The rarefaction curves obtained (Figure 1) gave an indication of the stability of the number of species in each sample. At the height of 30 m the curve has a tendency to stabilize with the number of 35 species, but at the ground level (0 m) an increase in the sampling effort causes the number of species in the curve to increase, without an observable limit. The species abundance indicated for each temporal sample has shown the largest number of specimens in the collections of the months of September and February.

 The estimates provide geometric models for the abundance curves; more flattened distributions correspond to more “diverse” samples [1]. We have observed some geometric models in our study, and they have shown that the temporal samples for April 06 and Sep. 05 had the highest diversity. As for the spatial samples (heights) in the strata of the forest, we found that the lower levels were more diverse (0 m and 8 m).

 In general, the dominant species was easily identified—*Culex portesi, *a total of about 84% of the specimens collected—and the rare species were dominant during night time collections. The diversity and richness of species was higher during daytime, when compared with night collections (not shown here).

 When taking the different heights as the sample (forest strata) the measures of diversity and richness have shown different results; the Berger-Parker and Shannon indices have indicated a greater diversity at ground level, while there was no difference between the indices at 8 m and 16 m; at 30 m the diversity was much smaller (*C. portesi*, the dominant species, was more abundant at the canopy level). The *t*-test for the Shannon index indicated a significantly higher diversity at the ground level, but no significant difference was found among the other three heights (8, 16, and 30 meters).

 In relation to the similarity, as measured by the Jaccard index, it was found that it decreases with the greater the distance (67%, 47%, and 39%) and was very high among the different neighbor strata.

 There were not clearly identified species ensembles in the different forest strata, probably due to the low diversity of species in some of them and also due to the fact that a few genera (*Culex*, *Haemagogus, Coquillettidia*,* and Sabethes*) seem to be highly adapted to the forest canopy (Confalonieri et al, in press [25]).

Studies of the relations between biological diversity and infectious disease risks have increased in the past few years; most of them point to an inverse relationship between the species richness and the increased risk of infection (“dilution effect”). However, most of theses studies did not include mosquito communities containing species vectors of pathogens [31–34]. We did not address infectious disease risk in this paper which is basically a contribution to the knowledge of the composition of mosquito communities in a region where more than 180 arbovirus species have been identified, 30 of them infecting humans [35].

Among the most frequent species found in this survey some are considered vectors of arboviral diseases endemic to the Amazon basin. This is the case of *Culex portesi*, the most frequent species, from which at least eleven types of arbovirus have been isolated [36]. Also three well-known vectors of yellow fever are included in this group of species (Table 1): *H. janthinomys, S. chloropterus*,* and H. leucocelaenus* [20]. *H. janthinomys* is also considered to be the main vector of Mayaro fever and from *Culex spissipes* the *Easter equine encephalitis* virus has been isolated; *Coquillettidia venezuelensis* is considered a secondary vector of *Oropouche* fever, a viral disease which causes periodic epidemics in the region. In the group of rare species, *Culex declarator* and *Sabethes belisarioi *can transmit the Saint Louis encephalitis virus, species of *Culex *(*Melanoconion*) are important vectors of Venezuelan Equine Encephalitis and *Culex pedroi* was found to harbor the *Eastern equine encephalitis* virus [35, 37–39].

## 5. Conclusions

This study, which is a complement to other published mosquito surveys of the same collecting site, shows comparatively the diversity of species in strata of a large sample of mosquito communities which includes several reported vectors of arboviral diseases, including yellow fever. Most of the mosquito community studies in the neotropics that have addressed diversity/similarity issues were developed outside the Amazon region and have compared the communities of vectors among different sites in a given locality or region and not different sampling strata of the same site, as was done here. The diversity studies involving different sites usually detect a relatively larger number of culicid species in the pooled samples which includes all sites, even with a much smaller number of specimens collected. In our site, the striking dominance of one single species (*C. portesi*, a carrier of the Mucambo and Saint Louis encephalitis viruses) has certainly influenced the estimation of some of the parameters to measure the diversity of species at different sampling strata. The fact that, in our study site, the largest mosquito species diversity was found at ground level, according to most of the estimators, may have implications for disease transmission since this is the level where humans are mostly exposed to mosquito bites. However, practically nothing is known about species interactions among neotropical mosquito species in specific communities and phenomena such as competition and displacement may determine dynamic changes in the composition of communities which would affect the vectorial capacity of the culicid populations.

 Future studies should address the issue of the possible relations between richness and diversity of populations of disease vectors and the risk of human infection, since most of the “dilution” studies have been so far concerned with the diversity of vertebrate hosts of pathogens. In this regard, it is necessary to clarify the role played by differences in the structure of mosquito communities in the determination of the risk of infectious disease transmission in specific ecosystems, such as the Amazon.

## Figures and Tables

**Table 1:** Culicid species collected at the LBA tower site in Caxiuanã, Melgaço, Pará, Brazil.

Species	Total specimens	Frequency (%)
*Culex portesi*	21341	83.911
*Coquillettidia venezuelensis*	831	3.267
*Haemagogus janthinomys*	814	3.201
*Culex spissipes*	521	2.049
*Sabethes chloropterus*	259	1.018
*Wyeomyia aporonoma*	213	0.837
*Haemagogus leucocelaenus*	173	0.680
*Culex adamesi*	170	0.668
*Wyeomyia sp.*	124	0.488
*Ochlerotatus serratus*	94	0.370
*Sabethes cyaneus*	84	0.330
*Culex sp.*	83	0.326
*Culex vomerifer*	71	0.279
*Culex (Melanoconion) sp.1*	56	0.220
*Limatus pseudometisticus*	56	0.220
*Coquillettidia albicosta*	50	0.197
*Ochlerotatus arborealis*	47	0.185
*Sabethes belisarioi*	44	0.173
*Culex spp.*	42	0.165
*Ochlerotatus agyrothorax*	42	0.165
*Sabethes glaucodaemon*	41	0.161
*Culex taeniopus*	33	0.130
*Culex (Microculex) sp.*	26	0.102
*Anopheles nimbus*	25	0.098
*Limatus flavisetosus*	22	0.087
*Coquillettidia arribalzagai*	21	0.083
*Sabethes tarsopus*	20	0.079
*Limatus durhamii*	18	0.071
*Sabethes amazonicus*	17	0.067
*Phoniomyia sp.*	14	0.055
*Culex declarator*	10	0.039
*Culex (carroli) sp.*	10	0.039
*Sabethes forattini*	9	0.035
*Culex (Melanoconion) sp.2*	8	0.031
*Anopheles mediupunctatus*	4	0.016
*Ochlerotatus fulvus*	4	0.016
*Chagasia bonneai*	3	0.012
*Culex pedroi*	3	0.012
*Ochlerotatus fulvithorax*	3	0.012
*Orthopodomyia fascipes*	3	0.012
*Tricoprosopon digitatum*	3	0.012
*Anopheles peryassui*	2	0.008
*Anopheles sp.*	2	0.008
*Haemagogus sp.*	2	0.008
*Ochlerotatus septemstriatus*	2	0.008
*Ochlerotatus ostator*	2	0.008
*Uranotaenia hystera*	2	0.008
*Wyeomyia melanocephala*	2	0.008
*Coquillettidia nigricans*	1	0.004
*Ochlerotatus scapularis*	1	0.004
*Sabethes sp.*	1	0.004
*Sabethes quasicyaneus*	1	0.004
*Uranotaenia calosomata*	1	0.004
*Psorophora albipes*	1	0.004

Total	25433	100

**Table 2:** Diversity indices for culicid specimens-temporal samples.

	Jul/05	Sep/05	Dec/05	Feb/06	Apr/06
Number of species *S *	34	33	25	31	41
Number of specimens *N *	3262	8236	3383	7349	3203
Shannon *H* ^'^	1.228	0.359	0.497	0.966	1.384
Evenness (*E* = *e* ^*∧*^ *H* ^'^/*S*)	0.101	0.043	0.066	0.085	0.097
Equitability *ε*	0.348	0.103	0.154	0.281	0.373
Berger-Parker	0.743	0.940	0.912	0.787	0.721

**Table 3:** Species richness estimates-temporal samples.

Month	*S*	*n*	*f1*	*f2*	*S*_chao1_	*S*_Bootstrap_	*S*_ACE_
July 05	34	3262	4	6	35.33	36.42	36.93
September 05	33	8236	7	8	36.06	36.74	38.33
December 05	25	3383	9	2	45.25	28.71	37.90
February 06	31	7349	4	1	39.00	32.75	32.69
April 06	41	3203	6	6	44.00	44.22	45.44

*S*: number of species; *n*: number of specimens; *f*1: number of *singletons*; *f*2: number of *doubletons*.

**Table 4:** Diversity indices for the different heights in the forest.

Heights	0 meters	8 meters	16 meters	30 meters
Number species *S*	38	34	34	33
Number Specimens *N*	4188	2394	4711	14140
Shannon *H* ^'^	1.517	1.046	0.988	0.469
Evenness (*E* = *e* ^*∧*^ *H* ^'^/*S*)	0.120	0.084	0.079	0.049
Equitability *ε*	0.417	0.297	0.280	0.134
Berger-Parker	0.667	0.785	0.785	0.918

**Table 5:** Student's *t*-test for the significant difference in diversity (Shannon) for the four heights.

Heights	Ground 8 m	8–16 m	16–30 m
*t*-Student	11.062	1.3467	1.3467
*P*	3.97*E*−28	0.18	0.18
Significance	Highly signif.	NS*	NS*

NS*: not significant (*P* > 0.05).

**Table 6:** Richness estimates to the forest strata (heights).

Height	*S*	*n*	*f1*	*f2*	*S*_chao1_	*S*_Bootstrap_	*S*_ACE_
0 meters	38	4188	6	4	42.50	40.95	43.85
8 meters	34	2394	7	4	40.13	37.41	37.61
16 meters	34	4711	13	2	76.25	39.06	53.83
30 meters	33	14140	6	1	51.00	35.74	38.12

*S*: number of species; *n*: number of specimens; *f*1: number of *singletons*; *f*2: number of *doubletons*.

**Table 7:** Jaccard similarity indices for the five different temporal samples.

Months	Jul. 05	Set. 05	Dec. 05	Feb. 06	Apr. 06
jul_05	1	0.619	0.439	0.625	0.551
set_05		1	0.513	0.625	0.652
dec_05			1	0.556	0.396
feb_06				1	0.622
apr_06					1

**Table 8:** Jaccard similarity indices for the four forest strata samples (heights).

Height	0 m	8 m	16 m	30 m
0 m	1	0.674	0.470	0.392
8 m		1	0.619	0.558
1 6 m			1	0.675
30 m				1
